# P-1162. Validation of a Maternal Sepsis Surveillance Definition Using Antimicrobial, Laboratory, and Clinical Criteria

**DOI:** 10.1093/ofid/ofaf695.1355

**Published:** 2026-01-11

**Authors:** W Wyatt Wilson, Lucy Fike, Marisa Young, Kiran M Perkins, Raymund Dantes

**Affiliations:** Emory University, Atlanta, GA; Centers for Disease Control and Prevention, Atlanta, Georgia; Emory University, Atlanta, GA; Centers for Disease Control and Prevention, Atlanta, Georgia; Emory University, Atlanta, GA

## Abstract

**Background:**

Infection, including sepsis, is a leading cause of maternal morbidity and mortality. Since a surveillance definition for maternal sepsis does not exist, U.S. burden estimates rely on healthcare billing data, which have historically provided inaccurate measurements of sepsis epidemiology in the adult population. Using mainly clinical criteria, we developed a maternal sepsis surveillance definition and validated it against existing clinical definitions of maternal sepsis.

**Figure:**
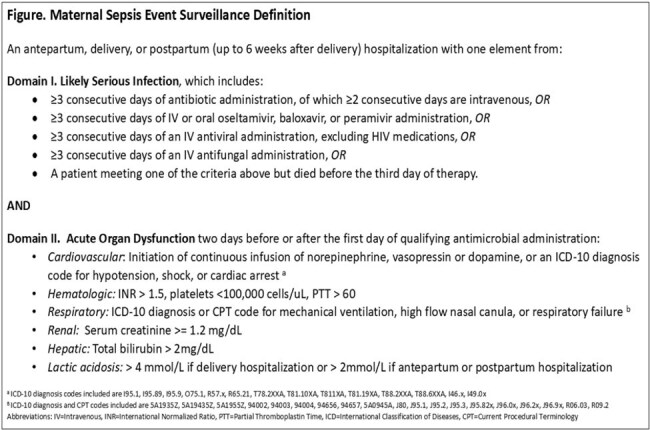

**Figure:**
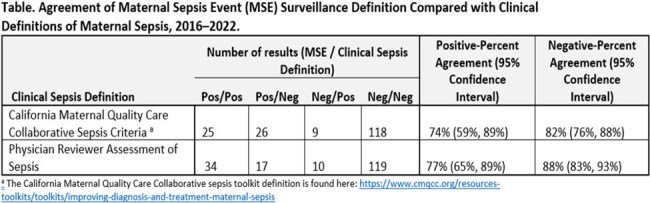

**Methods:**

We developed a Maternal Sepsis Event (MSE) definition incorporating antimicrobial, laboratory, and clinical criteria (Figure). We identified pregnancy and postpartum hospitalizations during 2016–2022 using billing data from two U.S. hospitals. Given the low prevalence of maternal sepsis, we then abstracted an enriched sample of hospitalizations with a Likely Serious Infection (LSI) (Figure). Patients receiving antimicrobials for infection prophylaxis only were excluded. After establishing interrater reliability (κ > 0.6), two physicians per hospital reviewed clinical and billing data in a random set of hospitalizations with a LSI to determine criteria for the MSE definition, California Maternal Quality Care Collaborative (CMQCC) clinical sepsis definition, and physician assessment of sepsis. We calculated descriptive statistics and positive- and negative-percent agreement (PPA, NPA) of the MSE definition.

**Results:**

Of 184 hospitalizations with a LSI, 51 (28%) met MSE criteria, 34 (19%) met CMQCC criteria, and 44 (24%) were deemed septic by physician assessment. Among patients with MSE, most were delivery hospitalizations (23, 45%); the most common organ dysfunction was lactic acidosis (21, 41%). The PPA, NPA, and false-positive rates of the MSE definition were 74%, 82%, and 18%, respectively, when compared to the CMQCC definition. When compared to the physician assessment, the PPA, NPA, and false-positive rates were 77%, 88%, and 12% respectively (Table).

**Conclusion:**

The MSE definition demonstrated a favorable NPA that minimizes false positives compared to existing clinical definitions, suggesting its utility as an accurate method for maternal sepsis surveillance. The MSE definition may be a useful tool to provide accurate estimates of the national maternal sepsis burden.

**Disclosures:**

All Authors: No reported disclosures

